# Nanostructured laminar tungsten alloy with improved ductility by surface mechanical attrition treatment

**DOI:** 10.1038/s41598-017-01458-0

**Published:** 2017-05-02

**Authors:** Hong-Yan Guo, Min Xia, Lap-Chung Chan, Kun Wang, Xiao-Xin Zhang, Qing-Zhi Yan, Man-Chao He, Jian Lu, Chang-Chun Ge

**Affiliations:** 1 State Key Laboratory for GeoMechanics and Deep Underground Engineering, China University of Mining and Technology, Beijing, 100083 China; 2 Institute of Nuclear Materials, University of Science & Technology Beijing, Beijing, 100083 China; 3 Department of Mechanical and Biomedical Engineering, City University of Hong Kong, Tae Chee Avenue Kowloon, Hong Kong 999077 China; 4 Laboratory for Nuclear Materials, Paul Scherrer Institute, 5323 Villigen PSI, Switzerland; 5 Center for Advanced Structural Materials, City University of Hong Kong Shenzhen Research Institute, 8 Yuexing 1st Road, Nanshan District Shenzhen, China

## Abstract

A nanostructured laminar W-La_2_O_3_ alloy (WL10) with improved ductility was prepared using a surface mechanical attrition treatment (SMAT). φ1.5 mm ZrO_2_ WL10 balls subjected to SMAT (called φ1.5 mm ZrO_2_ ball SMATed WL10) samples possess the best surface profile and excellent integrated mechanical properties (the ductile-brittle transition temperature (DBTT) value decreases by approximately 200 °C, and the bending strength decreases by 100 Mpa). A highly dense group of laminates was detected near the surface of the φ1.5 mm ZrO_2_ ball SMATed WL10 sample. The SMATed WL10 laminates were composed of a micro-grain layer, an ultrafine-grain layer and a nanosized-grain layer. The nanostructured laminar surface layer of the φ1.5 mm ZrO_2_ ball SMATed WL10 sample is approximately 1–2 μm. The top surface of the WL10 plates with and without the SMAT process possesses residual compressive stress of approximately −883 MPa and −241 MPa, respectively, in the *y* direction and −859 MPa and −854 MPa, respectively, in the *x* direction. The SMAT process could be a complementary method to further improve the toughness of tungsten-based materials.

## Introduction

Tungsten (W) and its alloys are now considered to be the most promising candidates for plasma facing materials (PFMs) in future fusion reactors because of the high melting point, high thermal conductivity, low deuterium/tritium retention and low sputter rates. However, these materials exhibit serious types of embrittlement, such as low-temperature brittleness, high-temperature or recrystallization brittleness and radiation-induced brittleness and hardness^1, 2^. Many methods have been developed to improve the performance of tungsten. Nanostructured (NS) W materials seem to be promising for nuclear applications since the large number of grain boundaries and dislocations in nanostructured W may result in better mechanical properties; in addition, grain boundaries could act as sinks for interstitial atoms, thus diminishing the degradation of the mechanical and thermal properties^3, 4^. Many methods, such as powder metallurgy (PM), severe plastic deformation (SPD) (including equal-channel angular extrusion (ECAE)^6, 7^ and high pressure torsion (HPT)^8^) and surface machining^9^, have been employed to refine the grains to ultrafine-grained (UFG) (>100 nm) or nanoscale sizes (<100 nm) to improve the mechanical properties or irradiation resistance performance of W^5^. However, nanostructures generally possess low ductility. Another method is to introduce extrinsic toughening mechanisms to prevent the propagation of cracks; these methods include reinforcement induced by laminates, fibres, whiskers, and particles, which primarily act behind the crack tip and locally shield it from the driving force^10, 11^. A solution to improve the ductility of tungsten or tungsten alloys is to fabricate laminar nanostructured materials that could deflect and re-nucleate cracks. Residual stress could often be induced in laminar structures during the fabrication process. Well-designed residual surface compression may prove to be extremely useful in inhibiting the growth of defects^12–14^.

In this study, we first apply the surface mechanical attrition treatment (SMAT) to bulk W–La_2_O_3_ (1 wt%) (WL10) to fabricate a nanostructured laminar tungsten alloy with improved ductility. WL10 is generally considered an important structural material in plasma facing components and has been shown to exhibit better mechanical properties, higher recrystallization temperature, and greater toughness than pure tungsten^1, 15^. SMAT is a manufacturing process that is based on the impact of spherical projectiles onto the sample surface^16, 17^ and is proposed as an effective method to achieve grain refinement down to the nanometre scale in the top surface layer; this method has been applied to many metals and alloys^16, 17^. Significant enhancements in the mechanical properties, such as tensile strength, fatigue limit, friction, and wear resistance, have been achieved with this method^18–20^. SMAT can be used to prepare nanostructure layers without an interface in bulk materials, resulting in a good bonding strength between surface layer and matrix. However, only a surface layer containing the nanocrystalline structure can be obtained using SMAT, and this method can only be used to process a sample with a flat surface of limited size^20^. In this manuscript, the extremely large strain and strain rate induced by the SMAT process were designed to store more dislocations in the intrinsic grains at room temperature, refine the grains into the nanograin size, generate a laminar structure, and induce residual stress; these properties are expected to improve the mechanical properties and irradiation resistance of WL10.

## Experimental Methods

**Figure 1:**
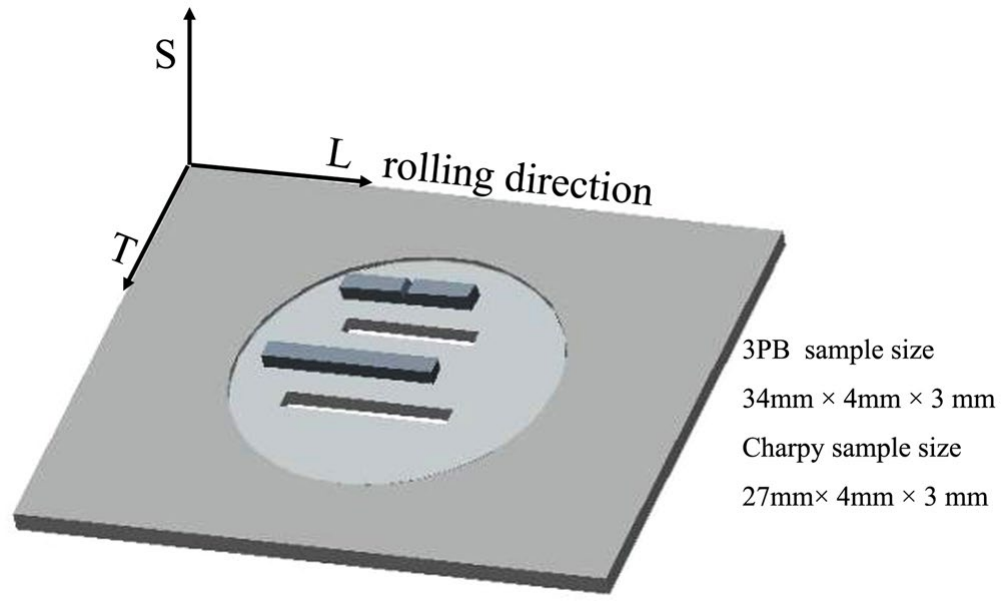
Cutting sketch of the specimens from SMATed WL10. Nomenclature of the sample orientation: “L” stands for longitudinal, “T” for transverse, and “S” for short transverse.

The material used in this investigation was a rolled WL10 plate (120 mm × 100 mm and 4 mm in thickness) of commercial purity (Beijing Tian-long Tungsten & Molybdenum Co., LTD). The setup and procedures of the SMAT process are described in the literature^21^. In this study, SMAT applied to WL10 was performed under vacuum at room temperature. Different balls (shots) with different diameters were used to study the SMAT process on WL10, and details of the SMAT parameters are described in Table 1. Both sides of each WL10 plate were treated. A cross-sectional TEM lamella was prepared using a focused ion beam (FIB) with a Zeiss Ultra 55. Transmission electron microscopy (TEM) observations were carried out on JEOL2010 and FEI Tecnai F20 microscopes operated at 200 kV. Three-point bending (3PB) specimens of 34 mm × 4 mm × 3 mm (length × width × height) were produced to measure the bending strength (see Fig. 1 for the cutting sketch of the specimens). Vickers microhardness tests were performed on the polished surface under a load of 200 g for 15 s. For the Charpy impact tests on specimens with dimensions of 27 mm × 4 mm × 3 mm (length × width × height), a notch depth of 1 mm and a notch root radius of 0.1 mm were created on the WL10 samples (see Fig. 1 for the cutting sketch of the specimens). All Charpy impact test samples were notched and prepared in the L-S direction^22^ (where “L” is longitudinal, which is in the rolling direction, and “S” is short transverse, which is the direction of the thickness of the plate). All samples were cut by electrical discharge machining. The specimens were heated to 673 K, 698 K, 723 K, 773 K, 873 K, and 973 K in an argon atmosphere and then pushed to the support outside the furnace and immediately hit by a striker (25 J) so that the samples were exposed to the air for a very short period. Because the contact time is short, the effects of air can be ignored. Three samples were tested for each temperature. Residual stresses were measured in the directions perpendicular to the SMATed surface of WL10 using a *μ*-X360n portable X-ray residual stress analyser (Pulstec Industrial Co., Ltd.)^23^.

**Table 1:** SMAT processes for the WL10 plate.

Ball	WC	Steel	ZrO_2_
Ball size/mm	φ2	φ3	φ1.5
Amplitude %	80	80	80
Time/min	30	30	30
Vibrating frequency/KHz	20	20	20

## Results and Discussion

### Optimization of the SMAT process

In the SMAT process, the velocity of the balls is a critical parameter in forming the nanostructures since it is significant in determining the strain-rate and localized strain of the material. Generally, a higher velocity results in a higher strain-rate, consequently enhancing the formation of nanostructures. Many parameters may affect the ball velocity in SMAT, such as the ball density, ball size and the power of the ultrasonic generator, as well as the mechanical properties (mainly the hardness) of the sample^24^.

**Figure 2:**
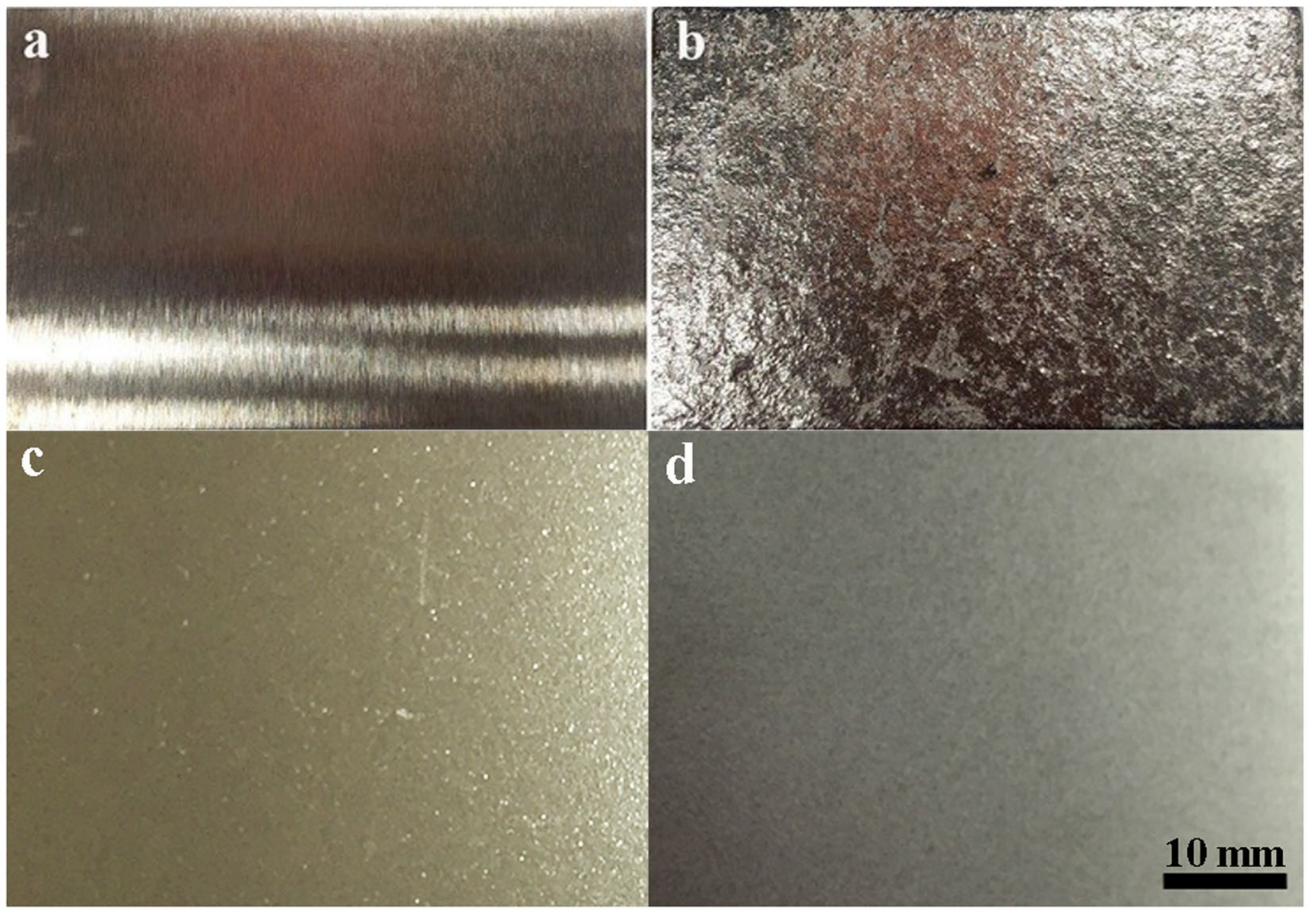
Surface profiles of WL10 that are (**a**) untreated, (**b**) WC ball treated, (**c**) ZrO_2_ ball treated, and (**d**) steel ball treated.

In this study, WC, steel, and ZrO_2_ balls with different densities were used to optimize the SMAT process for WL10. Figure 2 shows the surface profiles of WL10 before SMAT (2a) and after WC ball treatment (2b), ZrO_2_ ball treatment (2c), and steel ball treatment (2d). The WC ball SMATed WL10 sample possessed the highest surface roughness; therefore, the steel and ZrO_2_ ball-treated WL10 surface seem to be the most appropriate. The mass loss of different ball SMATed WL10 samples is shown in Table 2, revealing that the weight loss of the WC ball SMATed WL10 was up to 72 g (the most serious), which is consistent with the surface profile shown in Fig. 2(b). The surface profile and weight loss results indicated that the amount of energy transmitted by the WC balls is too high since lighter balls can attain a higher ceiling impact velocity. However, the amount of energy transmitted by a light ball is less than that of a ball with a larger mass. Thus, the WC ball can be eliminated for the WL10 in the SMAT process.

**Table 2:** Weight loss of different ball SMATed WL10 samples.

Parameter	WC	Steel	ZrO_2_
Ball size/mm	*φ*2	*φ*3	*φ*1.5
Δm/g	−72	−3	−3

“−” reference the SMATed sample mass loss.

**Figure 3:**
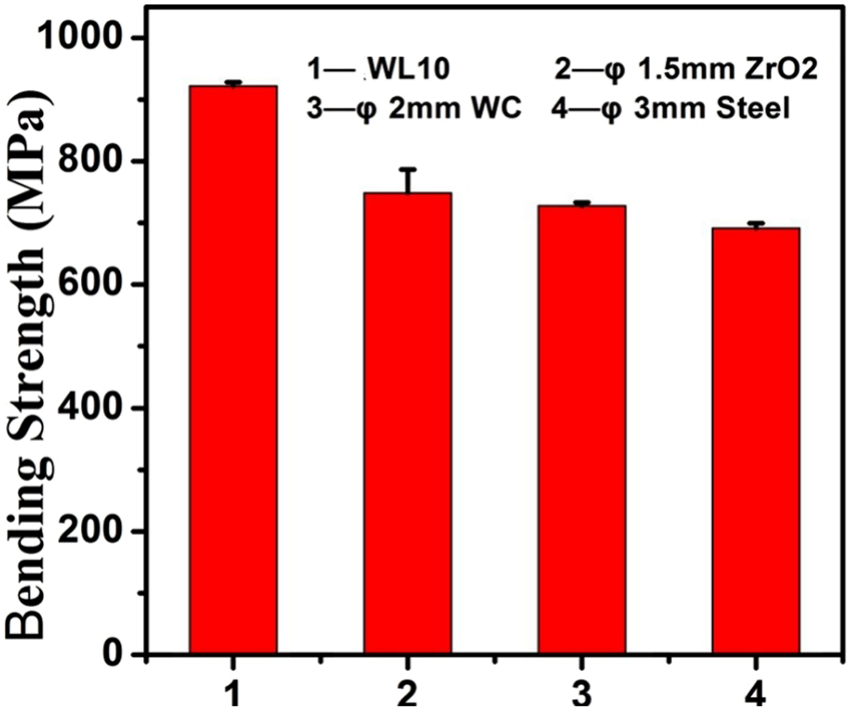
3PB bending strength of different ball SMATed WL10 samples.

Figure 3 shows the 3PB bending strength of the commercial WL10 (920 MPa), ZrO_2_ (760 MPa), WC (740 MPa), and steel ball (700 MPa) SMATed WL10 samples. A decrease in the bending strength was observed in all SMATed samples. In addition, the microhardness was tested in this work since it could reflect the relative density and the strain-hardening effects.

**Figure 4:**
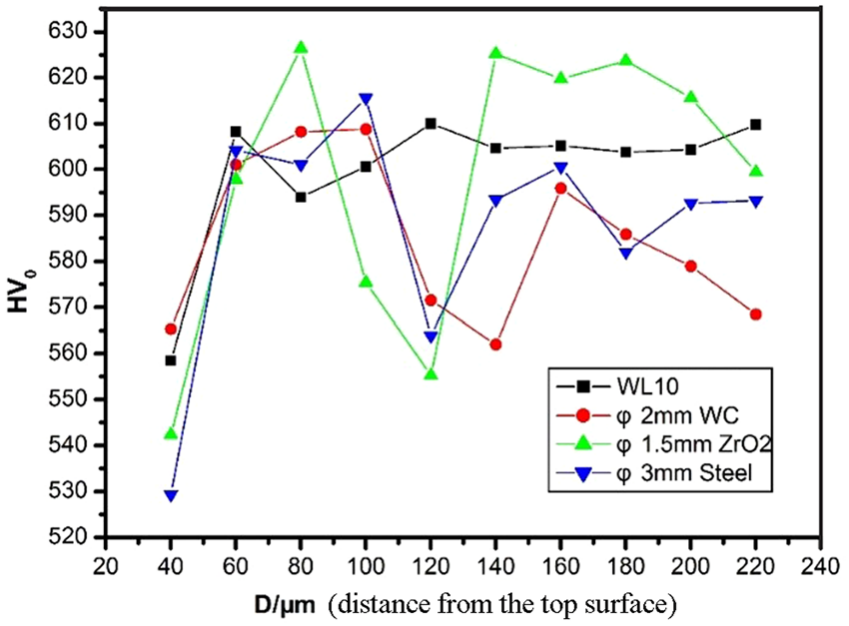
Microhardness of different samples taken from the cross-sectional plane in the depth direction. “D” references the distance beneath the top surface.

The microhardness distributions in the samples after SMAT was applied to various ball compositions are depicted in Fig. 4. As seen, the microhardness of the WL10 tested from the top surface to 60 *μ*m is lower than the mean value of the matrix, revealing that the surface may possess defects induced by machining. Additionally, all the SMATed samples possess the lowest microhardness at the positions of 120–140 *μ*m beneath the top surface, indicating the existing of microcracks. With microhardness positions above 140 *μ*m, the ZrO_2_ ball SMATed WL10 possesses a mean microhardness higher than that of the untreated WL10. In contrast, the WC and steel ball SMATed WL10 samples have a mean microhardness lower than that of the untreated WL10. These results indicate that the ZrO_2_ ball resulted in the best strain-hardening effect on the WL10. Since the ZrO_2_ ball has a smaller density than that of the WC steel ball, it can attain a higher velocity, and its sufficient weight enables a higher impinging energy to be carried to the sample surface^24^. Therefore, the ZrO_2_ ball could be more efficient than the WC steel ball. Based on the surface profiles and the 3PB bending strength and microhardness results, we selected the ZrO_2_ ball to further study the mechanical properties and microstructures of SMATed WL10.

### Mechanical properties and microstructure of the *φ*1.5 mm ZrO_2_ ball SMATed WL10

**Figure 5:**
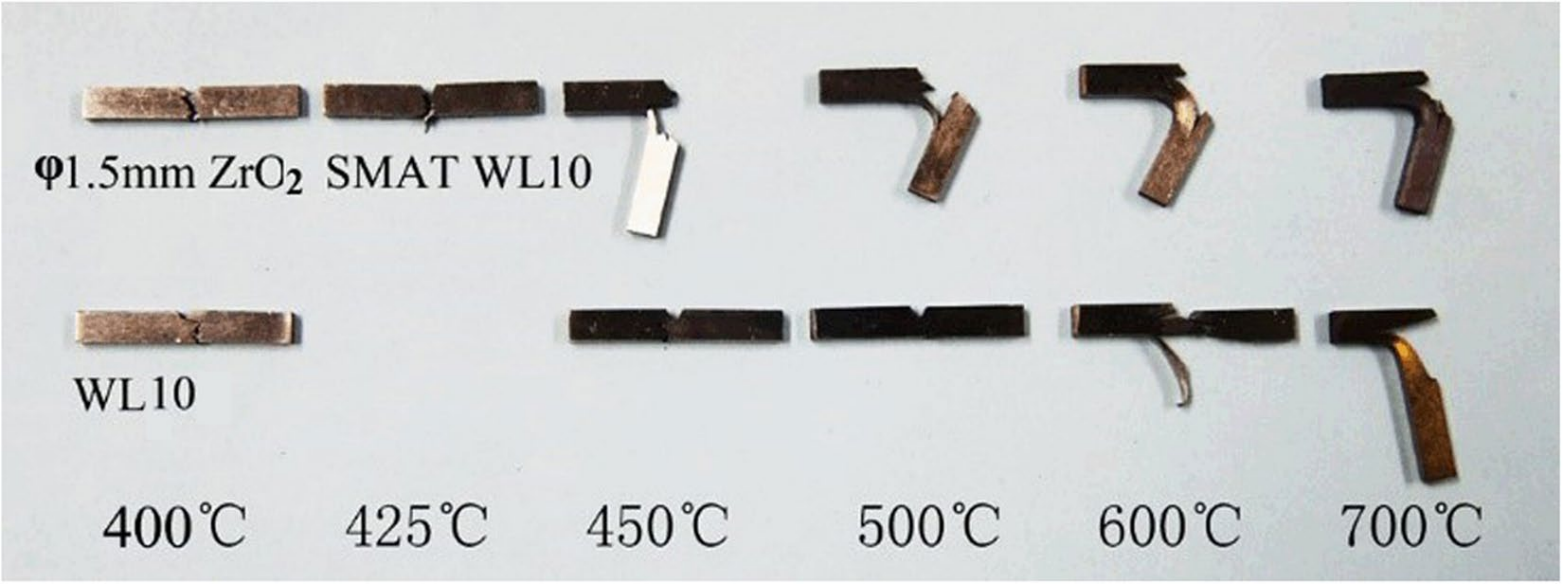
The Charpy impact test results of the WL10 and the *φ*1.5 mm ZrO_2_ ball SMATed WL10.

The ductility of the WL10 and the *φ*1.5 mm ZrO_2_ ball SMATed WL10 was characterized using Charpy impact tests. Since the commercial rolled tungsten sample was anisotropic and since the ductile-brittle transition temperature (DBTT) strongly depends on the orientation of the specimen, the Charpy curve may not be suitable to estimate the DBTT value^22^. In this case, the sample profile with fracture features was more acceptable to reveal the DBTT shift. The corresponding absorbed energy as a function of the test temperature of the WL10 and *φ*1.5 mm ZrO_2_ ball SMATed WL10 is listed in Table 3. Figure 5 shows the sample profile and reveals the appearance of the fractures. As seen, the estimated DBTT of the WL10 was approximately 973 K compared with the DBTT value (between 698 K and 723 K) of the *φ*1.5 mm ZrO_2_ ball SMATed WL10. Of particular interest, the fracture sample of the SMATed WL10 possesses a thin tail layer on the un-notched surface at 698 K, 723 K, and 773 K, which was different from the delamination feature from un-SMATed WL10 at 873 K and from the features listed in other reports. The SMATed sample at 773 K was almost fractured, except for the SMAT-induced thin layer connecting the two fractured pieces.

**Table 3:** The absorbed energy as a function of the test temperature of the WL10 and the *φ*1.5 mm ZrO_2_ ball SMATed WL10.

Test temperature & Samples	WL10 (Absorbed Energy, J)	*φ*1.5 mm ZrO_2_ ball SMATed WL10 (Absorbed Energy, J)
400 °C	0.375	0.875
425 °C	—	1
450 °C	0.375	1.75
500 °C	0.375	3.25
600 °C	0.375	5.25
700 °C	4.25	8.75

**Figure 6:**
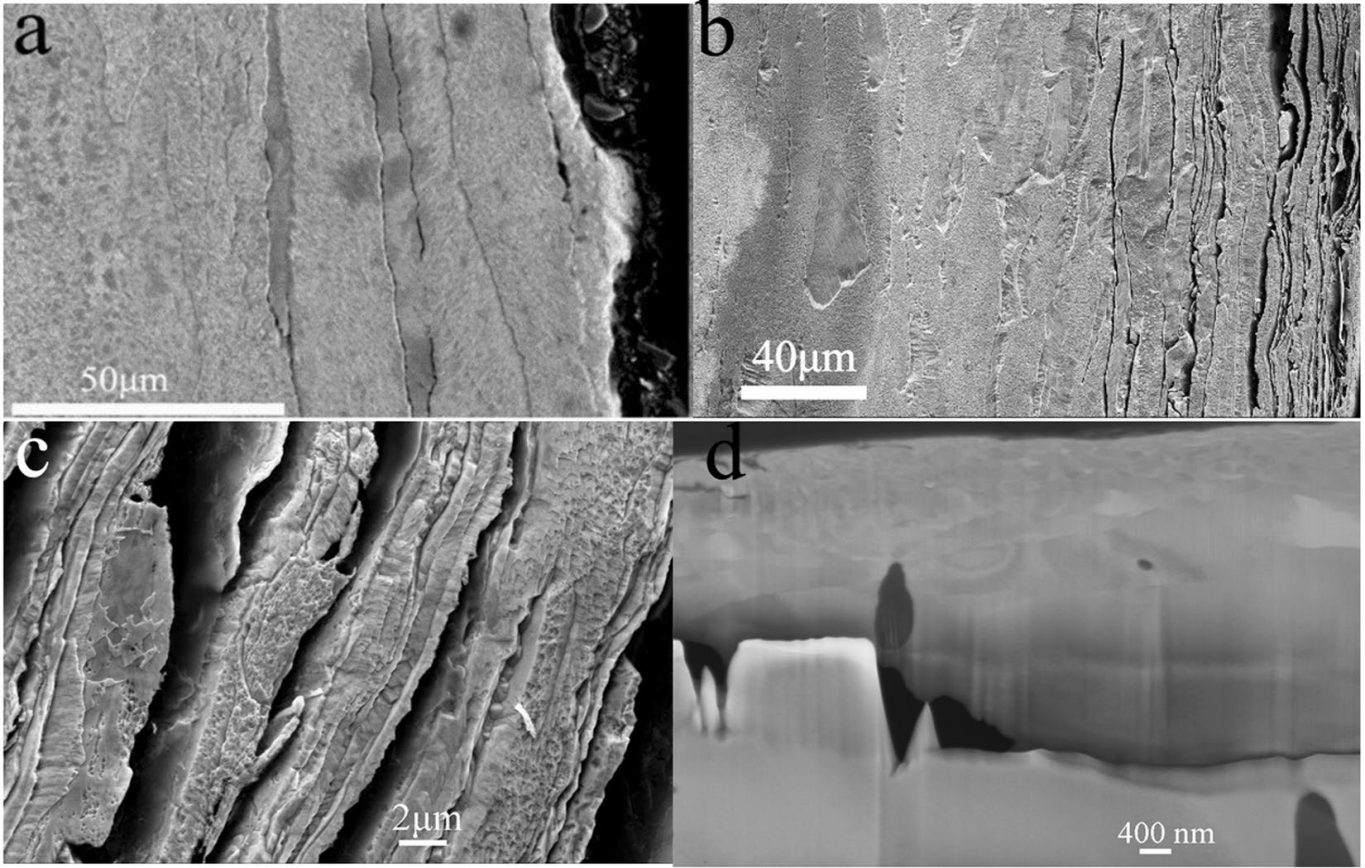
Cross sectional profile of WL10. (**a**) Untreated commercial WL10, (**b**) *φ*1.5 mm ZrO_2_ ball SMATed laminates observed near the surface, (**c**) enlarged SEM image of laminates in (**b**), and (**d**) cross sectional nanostructured surface layer taken from the FIB sample.

Figure 6(a) shows the cross-sectional SEM image of the untreated commercial WL10. Several microcracks were observed near the surface, which may be attributed to the rolling effect during the deformation process. For the *φ*1.5 mm ZrO_2_ ball SMATed WL10, as indicated in Fig. 6(b), a highly dense group of laminates was detected near the surface. The laminar structure is further demonstrated in Fig. 6(c) where the thickness of the individual laminate was approximately 1–2 *μ*m and the width was approximately 50 *μ*m. In addition, many microcracks were observed accompanying the laminates. Figure 6(d) shows the cross-sectional nanostructured surface layer taken from the FIB sample. As shown in the figure, after the SMAT process on the WL10 plate, a nanosized-grain layer with a mean grain size of approximately 50 nm and a neighbouring UFG layer with a mean grain size of less than 100 nm form on the surface with a total thickness of approximately 1–2 *μ*m in which the grain size gradually increases as a function of the depth beneath the surface. These results revealed that the SMATed WL10 laminates were composed of a micro-grain layer, a UFG layer and a nanosized-grain layer.

### TEM images of the top surface layer of the *φ*1.5 mm ZrO_2_ ball SMATed WL10

**Figure 7:**
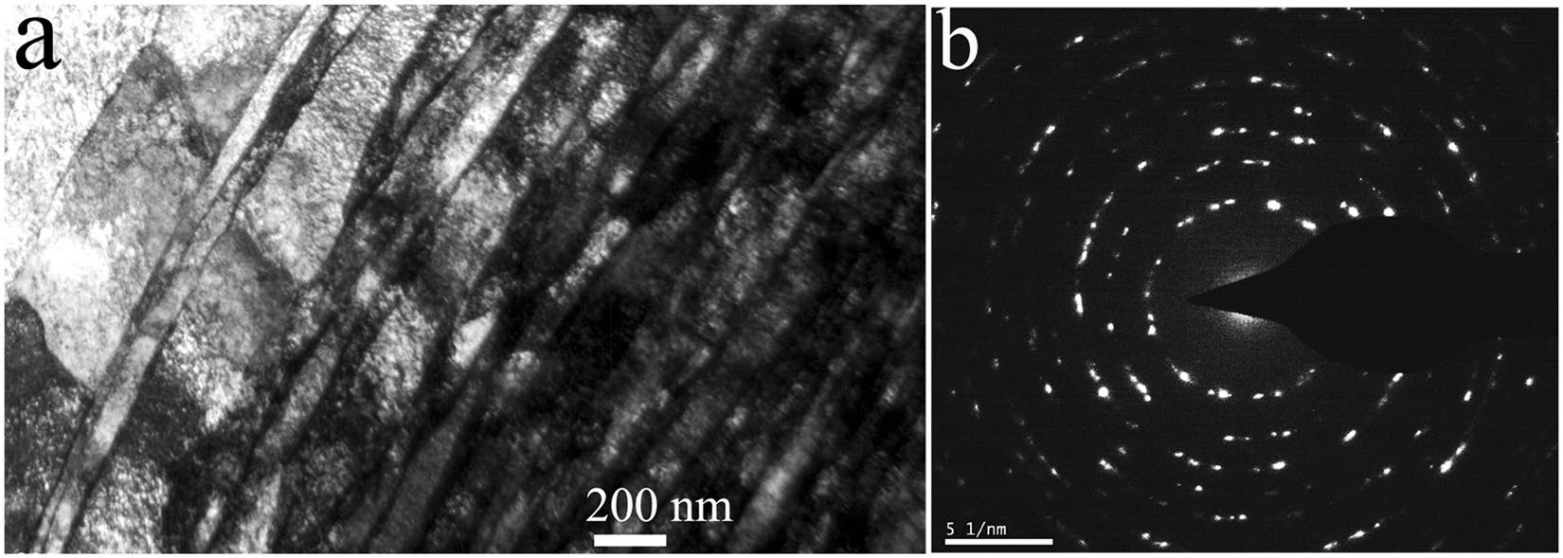
(**a**) Cross-sectional TEM micrograph of the *φ*1.5 mm ZrO_2_ ball SMATed WL10 and (**b**) the SAED pattern taken from (**a**).

**Figure 8:**
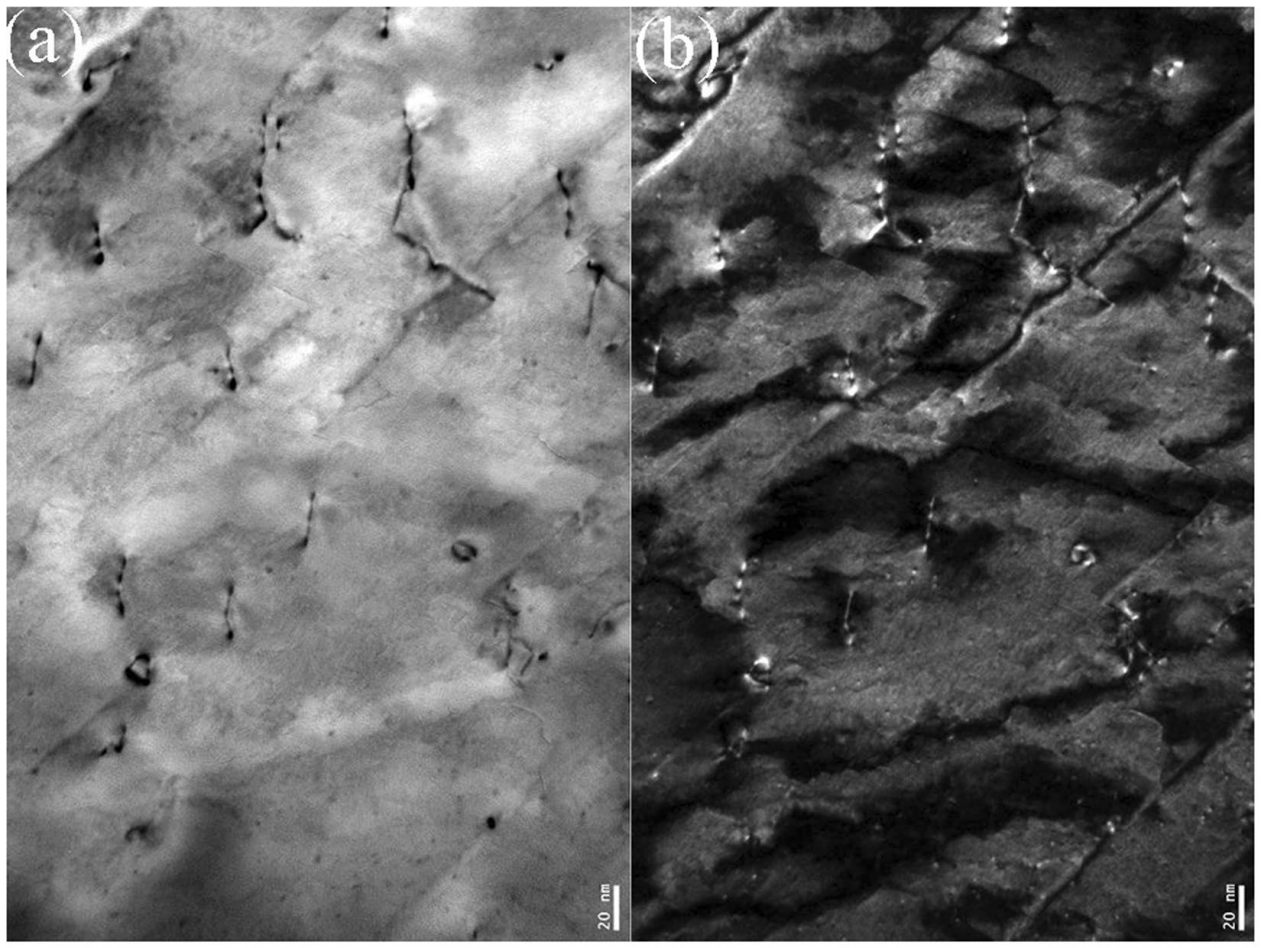
TEM images of dislocations and dislocation loops including (**a**) bright field and (**b**) dark field.

The nanostructured surface layer was further revealed through TEM characterization. Figure 7(a) shows the cross-sectional TEM micrograph of the *φ*1.5 mm ZrO_2_ ball SMATed WL10 sample, taken from the SMATed top surface, as indicated in Fig. 6(d). As seen in the figure, the gradient structure results from a gradual decrease in the applied strain with an increasing depth of the deformed layer, from the top surface to the substrate^25^. Accordingly, the grain size increases along the depth direction from the top surface to the substrate. Elongated W grains can be observed closest to the surface. Individual grains were prepared with sizes less than 50 nm and are separated by high-angle grain boundaries. Most of the grains are heavily strained and contain a high density of dislocations. The selected area electron diffraction (SAED) patterns of the SMATed layer in Fig. 7(b) are shown in circles, indicating the large fraction of high-angle grain boundaries. Additionally, some diffraction spots are split, which could be caused by the existence of low-angle grain boundaries. The azimuthal spreading of the diffraction spots is approximately 3–5°, indicating the existence of high internal strain. The high density of dislocations was further revealed in Fig. 8, as shown in the bright field and dark field TEM images of the W sample surface. The dislocation density of the W grains was approximately ~3.5 × 10^12^ cm^−2^. According to the microhardness results, the bending strength, cross-sectional profiles and micrographs, the decrease in the bending strength of the *φ*1.5 mm ZrO_2_ ball SMATed WL10 may contribute to the existence of microcracks in the laminar structure. However, in the case of the toughness of the *φ*1.5 mm ZrO_2_ ball SMATed WL10 sample, the given value of the DBTT in Fig. 5 should be lower (that is, the SMATed sample was tougher than the given data) since a notch depth of 1 mm and a notch root radius of 0.1 mm were created on the SMATed WL10 (the striker hit the side, which was cut first). Therefore, only one side contributed to the decrease of the DBTT in Fig. 5. We prepared un-notched Charpy test samples; however, the *φ*1.5 mm ZrO_2_ ball SMATed WL10 was so tough that even the striker (25J) could not destroy it at room temperature. Therefore, the SMAT process was expected to be effective in decreasing the DBTT of the WL10.

**Figure 9:**
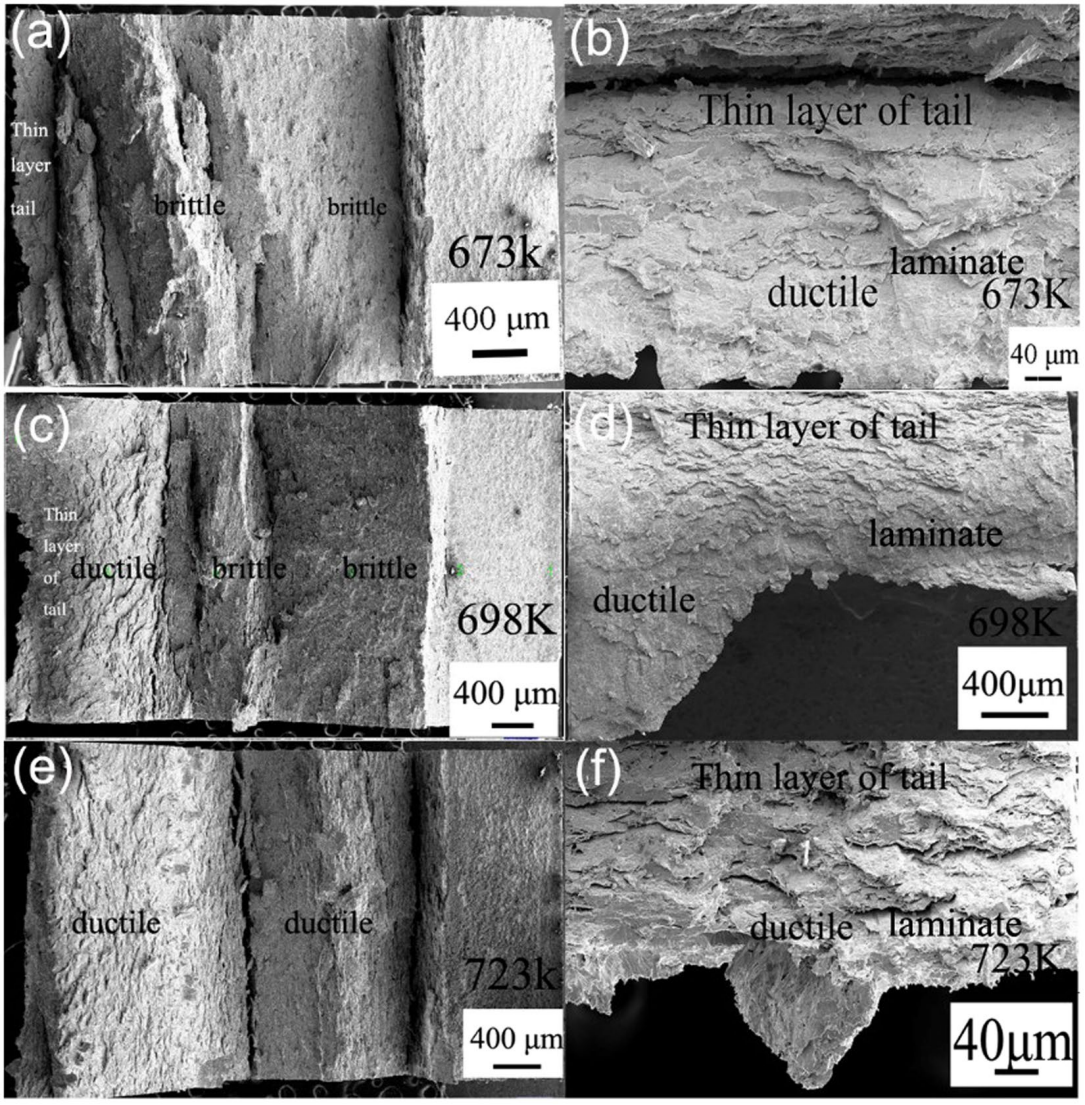
Fracture surfaces of the Charpy impact for the *φ*1.5 mm ZrO_2_ ball SMATed WL10; (**a**,**b**) 673 K, (**c**,**d**) 698 K, and (**e**,**f**) 723 K.

**Figure 10:**
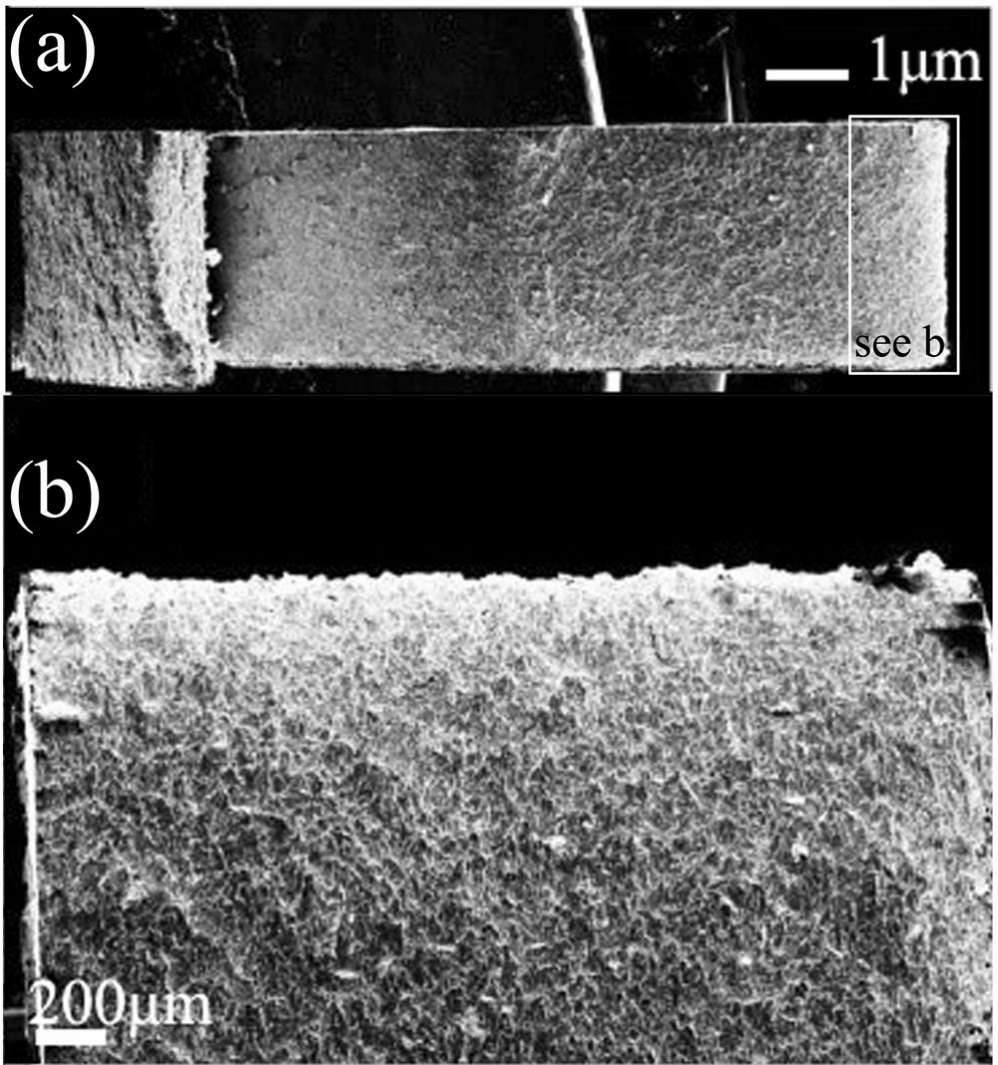
Fracture surfaces of the Charpy impact un-treated commercial WL10 sample at 873 K, where no laminate structure was observed.

To investigate the origin of the toughness improvements by the SMAT process, the microstructure of the ZrO_2_ ball SMATed and Charpy tested WL10 was observed. Figure 9 shows the fracture surfaces of due to the Charpy impact on *φ*1.5 mm ZrO_2_ ball SMATed WL10. When tested at 673 K, the sample exhibited an almost fully brittle cleavage fracture (see Fig. 9(a)). However, a very thin tail layer was observed on the un-notched and fractured surface, as shown in Fig. 9(b). When the temperature was increased to 698 K, the brittle fracture also dominated (Fig. 9(c)), but the length of the thin tail layer increased. Figure 9(e) shows the ductile fracture at 723 K, indicating that the DBTT is approximately 723 K. The thin tail layer was observed in both Figs 5 and 9(f). Of particular interest, apparent laminar structures were also observed in the SMATed thin tail layer of the fractured surfaces, as indicated in Fig. 9(b,d and f). In contrast, no laminar structure was detected in the un-treated commercial WL10, as indicated in the fracture surface at 873 K in Fig. 10(a and b).

**Figure 11:**
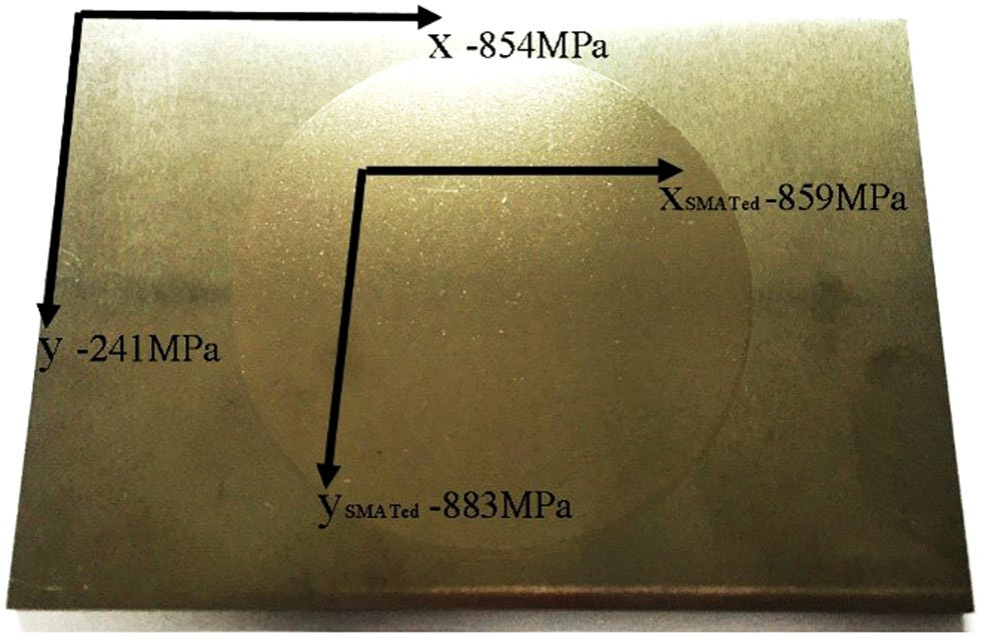
Residual stresses measured in the directions perpendicular to the SMATed surface (120 mm long in the × direction).

Meanwhile, during the grain refinement in the SMAT process, high compressive residual stresses could be simultaneously induced in the surface layer. In this case, a well-designed residual surface compression could be helpful in inhibiting the growth of defects^13, 14^. In this manuscript, the residual stress was measured for the specimens with and without SMAT, as shown in Fig. 11. The top surface of the WL10 plates with and without SMAT possesses a residual compressive stress of approximately −883 MPa and −241 MPa in the *y* direction, and −859 MPa and −854 MPa in the *x* direction, respectively. The residual compressive stress in the *x* direction with and without SMAT exhibited similar values, and the residual compressive stress in the un-treated surface may be attributed to the deforming and rolling effect of the WL10 plate. However, in the *y* direction, the residual compressive stress was significantly improved using the SMAT process.

The results revealed that the SMAT process could be used to prepare nanostructured laminar tungsten alloys with improved ductility, and a high compressive residual stress could be simultaneously induced on the surface layer. However, the origin of the good ductility was manifold due to the multi-scale-based deformation mechanisms and fracture mechanisms integrated into the laminates. The following two major factors contributed to the ductility improvements of the SMATed WL10 sample.

First, the micro-grain layer in the laminate plays a primary role in improving the ductility since its ability to deform plastically was conditioned by the dislocations. In addition, a large fraction of high-angle grain boundaries at the top surface was induced by the SMAT process (as indicated by the SAED pattern in Fig. 7(b)), which could lead to a high strain-hardening rate in the UFG layer, consequently, leading to a high total elongation^12, 26^.

Second, the residual stress strongly affects the mechanical properties and the selection of the crack path in the brittle laminates of ceramic-like materials^13, 14^. A well-designed residual stress profile on the surface of the material and a greater initial compression to a depth slightly below the surface can impede the surface crack propagation. This process toughens the materials and leads to multiple cracking, which serves as a forewarning of the final failure^27^. As indicated in Fig. 11, a high compressive residual stress was induced in the nanosized-grain layer, and multiple cracks were observed at the fracture surface, as shown in Fig. 6. Thus, most of the cracks were arrested in the nanosized-grain thin tail layer. The existence of the compressive residual stress in the SMAT layer could impede advancement of the crack along the direction perpendicular to the interface plane when the first crack was initiated. Therefore, the toughening mechanisms induced by the compressive residual stress and the multiple parallel crack shielding effects greatly improve the ductility of SMATed WL10^12^.

Table 4 gives a comparison between the present and previously reported data related to the DBTT of tungsten-based materials. Generally, the DBTT of tungsten-based materials decreases by alloying, particle strengthening, and working processes such as rolling, forging, and injection moulding. All of these methods have been demonstrated to effectively improve the ductility of tungsten-based materials. In the case of SMAT processing in this report, the DBTT of the SMATed WL10 sample is approximately 723 K (intuitively seen in Fig. 5 and Table 4), which is much lower than that of the untreated bulk WL10 in this report at 973 K^16^. In this sense, the SMAT process could be a complementary method to further decrease the DBTT value of tungsten-based materials.

**Table 4:** Comparison between the present and previously reported data related to the DBTT of tungsten-based materials.

Material/Size	Working process	DBTT(K)	Dimension(mm)	Method	Ref.
WL10	Swaging + Rolling + SMAT	≤723	27 × 4 × 3	Charpy	our
WL10	Swaging + Rolling	973	27 × 4 × 3	Charpy	our
WL10	Swaging + Rolling	973	10 × 10 × 55	Charpy	28
W − 0.5 ZrC (8.5 mm thick plate)	Rolling	373	2 × 2 × 20	3PB	29
W − 2 Y_2_O_3_ (2 mm thick, *Φ* 95 mm)	Hot Forging	473	2 × 2 × 25	3PB	30
Pure W (10 mm thick plate)	Rolling	473	2 × 4 × 20	3PB	31
Pure W (4 mm thick)	HIP	473	2 × 3.3 × 20	3PB	31
W − 0.2 TiC (1 mm thick)	Forging + Rolling	440	1 × 1 × 20	3PB	32
W − 0.25 Ti−0.05 C (1 mm thick plate)	Rolling	260	1 × 1 × 20	3PB	32
W − 1% Y_2_O_3_	Injection molding	1273	3 × 4 × 27	Charpy	33
Pure W	Injection molding	1173	3 × 4 × 27	Charpy	33
W − 0.5 TiC	HIP + Forging	484	1 × 1 × 20	3PB	34
Pure W (0.1 mm thick foil)	Rolling + Joining	373	4 × 15 × 33	Charpy	35

## Conclusions

Nanostructured laminar WL10 was prepared using a surface mechanical attrition treatment (SMAT). A ZrO_2_ ball has a smaller density than that of a WC steel ball; therefore, it can attain a higher velocity, and its sufficient weight enables a higher impinging energy to be carried to the sample surface. The *φ*1.5 mm ZrO_2_ ball SMATed WL10 sample possesses the best surface profile. A highly dense group of laminates was detected near the surface of the *φ*1.5 mm ZrO_2_ ball SMATed WL10 sample. The SMATed WL10 laminates were composed of a micro-grain layer, an ultrafine-grain layer and a nanosized-grain layer. The nanostructured laminar surface layer of the *φ*1.5 mm ZrO_2_ ball SMATed WL10 sample is approximately 1–2 *μ*m. The bending strength of the SMATed WL10 was attributed to the microcracks in the laminates. The top surface of the WL10 plates with and without the SMAT process possesses residual compressive stresses of approximately −883 MPa and −241 MPa, respectively, in the *y* direction, and −859 MPa and −854 MPa, respectively, in the *x* direction. The existence of the micro-grain layer in the laminate, the compressive residual stress and multiple parallel crack shielding (laminates) were considered to be the three major factors that contribute to the ductility improvements of the SMATed WL10. The SMAT process could be a complementary method to further improve the toughness of tungsten-based materials.
