# Author Correction: Nanostructured laminar tungsten alloy with improved ductility by surface mechanical attrition treatment

**DOI:** 10.1038/s41598-018-25323-w

**Published:** 2018-04-27

**Authors:** Hong-Yan Guo, Min Xia, Lap-Chung Chan, Kun Wang, Xiao-Xin Zhang, Qing-Zhi Yan, Man-Chao He, Jian Lu, Chang-Chun Ge

**Affiliations:** 1 State Key Laboratory for GeoMechanics and Deep Underground Engineering, China University of Mining and Technology, Beijing, 100083 China; 2 Institute of Nuclear Materials, University of Science & Technology Beijing, Beijing, 100083 China; 3 Department of Mechanical and Biomedical Engineering, City University of Hong Kong, Tae Chee Avenue Kowloon, Hong Kong 999077 China; 4 Laboratory for Nuclear Materials, Paul Scherrer Institute, 5323 Villigen PSI, Switzerland; 5 Center for Advanced Structural Materials, City University of Hong Kong Shenzhen Research Institute, 8 Yuexing 1st Road, Nanshan District, Shenzhen China

Correction to: *Scientific Reports* 10.1038/s41598-017-01458-0, published online 02 May 2017

The original version of this Article omitted an affiliation for Jian Lu. The correct affiliations are listed below:

Department of Mechanical and Biomedical Engineering, City University of Hong Kong, Tae Chee Avenue Kowloon, Hong Kong 999077, China.

Center for Advanced Structural Materials, City University of Hong Kong Shenzhen Research Institute, 8 Yuexing 1st Road, Nanshan District, Shenzhen, China.

In addition, the Acknowledgements section in this Article contained errors:

“The authors gratefully acknowledge the financial support of the ITER-National Magnetic Confinement Fusion Program (2010GB109000 and 2014GB123000). J. LU acknowledges the financial support provided by the MOST 973 Program of China under the grant 2012CB932203 and the Croucher Foundation Grant (CityU9500006).”

now reads:

“The authors gratefully acknowledge the financial support of the ITER-National Magnetic Confinement Fusion Program (2010GB109000 and 2014GB123000). J. LU acknowledges the financial support from Guangdong Science and Technology Department (Ref: 2014B050504003) and SZSTI (Ref: ZDSYS201602291653165).”

These errors have now been corrected in the HTML and PDF versions of this Article.

